# Supplementary material for: Changes in the place of death before and during the COVID-19 pandemic in Japan
Source: PLoS One. 2024 Feb 28;19(2):e0299700. doi: 10.1371/journal.pone.0299700 (PMC10901324; doi:10.1371/journal.pone.0299700)
Supplement: S1 Table — (DOCX) [file pone.0299700.s001.docx]

| Age/facility | 2001 | 2021 |
| --- | --- | --- |
|  | No. of deaths (%) | No. of deaths (%) |
| 0–19 years |  |  |
| Home | 817 (9.5) | 689 (17.9) |
| Hospital | 7090 (82.4) | 2811 (72.9) |
| Nursing home | 0 (0.0) | 1 (0.0) |
| 20–64 years |  |  |
| Home | 27746 (14.6) | 36296 (29.9) |
| Hospital | 145183 (76.6) | 76467 (63.0) |
| Nursing home | 157 (0.1) | 1050 (0.9) |
| ≥65 years |  |  |
| Home | 102761 (13.3) | 210904 (16.0) |
| Hospital | 635904 (82.4) | 891585 (67.8) |
| Nursing home | 24312 (3.2) | 193646 (14.7) |
